# Effects of Visual Scene Complexity on Neural Signatures of Spatial Attention

**DOI:** 10.3389/fnhum.2020.00091

**Published:** 2020-03-24

**Authors:** Lia M. Bonacci, Scott Bressler, Jasmine A. C. Kwasa, Abigail L. Noyce, Barbara G. Shinn-Cunningham

**Affiliations:** ^1^Department of Biomedical Engineering, Boston University, Boston, MA, United States; ^2^Graduate Program in Neuroscience, Boston University, Boston, MA, United States; ^3^Department of Psychology, Carnegie Mellon University, Pittsburgh, PA, United States; ^4^Neuroscience Institute, Carnegie Mellon University, Pittsburgh, PA, United States

**Keywords:** EEG, visual spatial attention, alpha oscillations, evoked potential, scene complexity

## Abstract

Spatial selective attention greatly affects our processing of complex visual scenes, yet the way in which the brain selects relevant objects while suppressing irrelevant objects is still unclear. Evidence of these processes has been found using non-invasive electroencephalography (EEG). However, few studies have characterized these measures during attention to dynamic stimuli, and little is known regarding how these measures change with increased scene complexity. Here, we compared attentional modulation of the EEG N1 and alpha power (oscillations between 8–14 Hz) across three visual selective attention tasks. The tasks differed in the number of irrelevant stimuli presented, but all required sustained attention to the orientation trajectory of a lateralized stimulus. In scenes with few irrelevant stimuli, top-down control of spatial attention is associated with strong modulation of both the N1 and alpha power across parietal-occipital channels. In scenes with many irrelevant stimuli in both hemifields, however, top-down control is no longer represented by strong modulation of alpha power, and N1 amplitudes are overall weaker. These results suggest that as a scene becomes more complex, requiring suppression in both hemifields, the neural signatures of top-down control degrade, likely reflecting some limitation in EEG to represent this suppression.

## 1. Introduction

At any given moment, the external world may present us with a multitude of rapidly changing stimuli. Despite the complex dynamics of visual input, we are able to effortlessly pick out a single object against a background of irrelevant distraction. Not only can we select this object, but we can also sustain attention to it, tracking how it changes or moves over time (e.g., locating a friend in a crowd and tracking their position as you attempt to grab their attention). In order for this selection and tracking to be successful, our perception of target stimuli must be enhanced while that of distractor stimuli is suppressed (James, 1890), presumably through enhancement or suppression of their respective neural representations. Many studies of visual attention provide evidence for neural mechanisms that both enhance target and suppress distractor stimuli in complex scenes, but very few consider these processes during attention to dynamic stimuli (but see for example Agam and Sekuler, 2007; Drew et al., 2009; Song and Nakayama, 2009; Kerr et al., 2011; Payne et al., 2013; Stormer et al., 2013; van Ede et al., 2017). Furthermore, many of these studies focused on sustained attention to a single stimulus. In order to understand both attentional enhancement and attentional suppression in the presence of various configurations of dynamic distracting stimuli, we systematically manipulated scene complexity while subjects monitored a visual stimulus during three separate experiments.

In vision, attention is often deployed to a spatial location, either via bottom-up guided cues or via top-down volitional (endogenous) direction of attention (Posner, 1980; Posner et al., 1980; Rosen et al., 2014; Huffman et al., 2018; Wolfe and Utochkin, 2019). Once a location is attended, visual features at that location, such as color, shape, and orientation, are integrated and perceived as belonging to whole objects (Treisman and Gelade, 1980; Shinn-Cunningham, 2008; Humphreys, 2016). Spatial selective attention can greatly affect our perception of complex scenes. For example, in a complex scene, if attention is directed away from a particular location, large changes in objects at this location can go completely unnoticed even if the location is clearly visible within our visual field (Simons and Chabris, 1999; Simons and Rensink, 2005; Drew et al., 2013). The neural mechanisms that implement such endogenous control of spatial attention are still not well understood.

Evidence of top-down attentional processes can be found using electroencephalography (EEG). One common EEG measure is an evoked response, or event-related potential (ERP), which reflects ensemble neural firing that is phase locked to stimulus events. One ERP component that is often used as an index of sensory processing is the N1, a large negative deflection of the ERP that occurs 100–200 ms after stimulus onset. N1 responses are elicited by both auditory events (Näätänen and Picton, 1987) and visual events (Mangun and Hillyard, 1991), and can be modulated by endogenous top-down attention (Hillyard and Anllo-Vento, 1998; Choi et al., 2013, 2014). Deploying attention to a specific item or location enhances the N1 elicited by the attended object (Hillyard and Anllo-Vento, 1998; Choi et al., 2014); revoking attention from a distractor may reduce the corresponding N1 (Choi et al., 2013). N1 amplitude thus provides one index of attentional modulation in sensory processing.

An additional EEG marker of top-down attentional control is the induced EEG response. Unlike the evoked response, induced activity is not phase locked to stimulus events; instead its power is loosely related to event timings Kalcher and Pfurtscheller (1995). In particular, oscillations in the alpha band (8–14 Hz) have been associated with selective attention in both vision and audition (Sauseng et al., 2005; Klimesch et al., 2007; Kerlin et al., 2010; Banerjee et al., 2011; Payne et al., 2013; Payne and Sekuler, 2014; van Diepen et al., 2016). Unlike the N1, alpha power decreases in regions of cortex that are processing an attended or task-relevant stimulus, and increases in regions that represent distractors or irrelevant locations (Worden et al., 2000; Payne et al., 2013; Payne and Sekuler, 2014). Thus, alpha oscillations are thought to be associated with a top-down suppression mechanism (Kelly et al., 2006; Foxe and Snyder, 2011; Payne and Sekuler, 2014; Zumer et al., 2014), facilitating selection of relevant objects by attenuating neural processing of irrelevant objects.

While the N1 response has been studied extensively as an index of top-down attention, the characteristic behavior of alpha activity is less clear. Many studies have shown that modulation of alpha power following a spatial cue reflects anticipatory biasing of attention to a specific location (Worden et al., 2000; Thut et al., 2006). Other studies have shown that this modulation persists following brief presentation of a single stimulus (Sauseng et al., 2005; van Diepen et al., 2016). However, very few studies (Kelly et al., 2006; Händel et al., 2011; Keitel et al., 2019) have examined the role of alpha oscillations during sustained attention to dynamic target stimuli.

Because the external world is full of dynamically changing sensory input, it is important to understand how the brain performs selection over time when sustained attention to shifting stimuli is required. Furthermore, the external world rarely presents us with only two competing, yet spatially separated objects at a time. Rather, we are often tasked with ignoring many objects at once, which may or may not occupy space close to the target. To understand how alpha oscillations relate to attentional focus in everyday visual processing, it is important to test conditions that more closely mimic these attributes.

In this study, we designed a selective attention paradigm in which subjects were required to track a visual object as it changed over time. By adjusting the number of locations in which irrelevant stimuli appeared, we created three experiments, each with different scene complexity. Using EEG, attentional modulation of both the N1 response and induced alpha power were measured and compared across these three paradigms. We had two goals. First, we wished to confirm that the N1 and alpha power reflect enhancement and suppression during visual attention to dynamic stimuli. Second, by comparing these measures across tasks of increasing scene complexity, we hoped to shed light on how the EEG representation of top-down control changes when subjects are tasked with suppressing an increasing number of irrelevant stimuli. We hypothesized that attention would continuously modulate both the N1 and alpha power over time, but that the degree of this modulation would vary depending on the number of irrelevant stimuli present.

## 2. Methods

### 2.1. Subjects

Data from a total of 31 subjects with normal or corrected-to-normal vision and no known neurological disorders were analyzed in this study—10 for Experiment 1 (4 male, mean age = 26, *SD* = 5.83), 11 for Experiment 2 (6 male, mean age = 21.64, *SD* = 3.38), and 10 for Experiment 3 (3 male, mean age = 22.54, *SD* = 2.87). Two additional subjects were recruited for each of Experiments 2 and 3, but their data were discarded due to too many noisy or incorrect response trials. Subjects were recruited primarily from the Boston University student population and gave written consent before participating. Compensation was given in the form of a base pay rate in addition to a bonus for each correct response during the task ($ 0.02; up to $7.50 per hour). All procedures were approved by the Boston University Institutional Review Board.

### 2.2. Experimental Task and Stimuli

Three different experiments were designed, each with an increasing number of distractors appearing on screen. By increasing the amount of irrelevant, or to-be-ignored stimuli, we manipulated attentional demands, requiring greater top-down control of attention to successfully complete the task.

**Figure 1:**
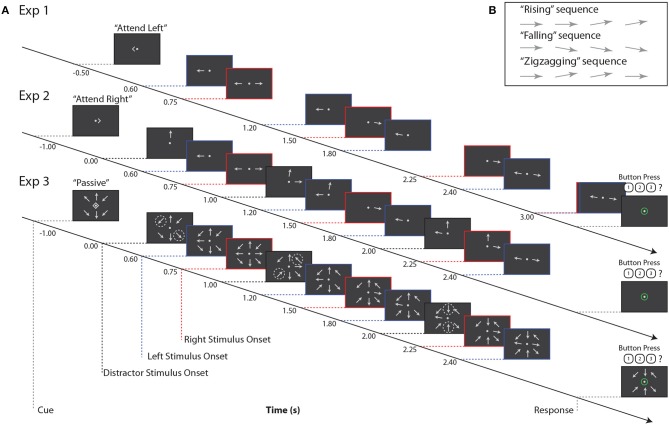
**(A)** Example stimuli for all three experiments. Red, blue, and black dashed lines indicate right, left, and distractor stimulus onsets, respectively. Note that for Experiment 3, black dashed lines indicate times at which pairs of arrows at distractor locations change orientation, as indicated by the dashed circles. These circles are for illustrative purposes only, and did not appear during the experiment. **(B)** Example trajectories for target stimuli. Note that if a sequence contained three onsets (not pictured here), arrow orientation changes always occurred at the second onset.

In all three experiments, subjects tracked the orientation trajectory of an “arrow,” that is, a line with a triangular arrowhead affixed to one end, over a short sequence of onsets and offsets (Figure 1). The arrowhead was always on the more peripheral end of the target line. After each onset, the arrow remained on the screen for 0.3 s, followed by an interstimulus interval that depended on the location of the target arrow (either 0.3 or 0.45 s). The arrow was always horizontal at the first onset, and at some subsequent onset it rotated by 10° to point slightly upwards or downwards. The arrow could remain in this new orientation for the remaining onsets that made up the trial, or it could revert to the horizontal orientation. Subjects were asked to categorize orientation trajectories as “rising,” “falling,” or “zigzagging” (Figure 1B); these categories were equiprobable, and were chosen independently for each stimulus. Subjects reported the perceived category via keypress.

On each trial, subjects were instructed to maintain fixation on a central dot and refrain from blinking or closing their eyes. Each trial began with a visual cue indicating “attend left,” “attend right,” or “passive”; these trial types were equiprobable. Subjects were instructed to attend to that single arrow while ignoring all other stimuli. On passive trials, subjects were to ignore all stimuli and refrain from responding. For all three experiments, there was an interval of 1.5–1.7 s between the end of the response period and the start of the next trial.

Stimuli were generated and presented using custom MATLAB (The Mathworks, Inc., Natick, MA) software with the Psychtoolbox 3 extension (Brainard, 1997; Kleiner et al., 2007); responses were recorded on each trial.

#### 2.2.1. Experiment 1

In Experiment 1, two arrows were shown on each trial, one to the left and one to the right of a central fixation point (approximately 4.8° eccentricity; Figure 1A). Subjects were cued to report the orientation trajectory of either the left or right arrow; the non-cued arrow served as the sole distractor.

The orientation trajectories of each arrow stimulus comprised either four (the lagging sequence) or five (the leading sequence) onsets and offsets on each trial. Leading and lagging sequences were equiprobable on the left or right; for comparison with subsequent paradigms, which always presented leading stimuli on the left, we here analyze only trials in which left stimuli were leading and right stimuli were lagging. The onset times of left and right arrows were purposely staggered to allow us to isolate neural responses to stimuli in the time domain (Figure 1A). In Experiment 1, subjects completed 360 trials; because we only analyzed left-leading trials, data presented below are from 180 trials.

#### 2.2.2. Experiment 2

Experiment 2 was similar to Experiment 1, with the addition of a third, always-ignored distractor arrow stimulus, presented at approximately 4.8° visual angle above the central fixation point (Figure 1A). This third stimulus always had 3 onsets, staggered in time from those of the left and right arrows, and its orientation trajectory was generated as in the other stimuli, but tilting left or right rather than upward or downward.

Orientation trajectories in this experiment were statistically identical to those in Experiment 1, except that, for simplicity, the left sequence always led the right sequence in time, and left and right sequences had 4 and 3 arrow onsets, respectively. Note that while the number of onsets in each sequence was lower than in Experiment 1, which had 5 and 4 left and right arrow onsets, respectively, the interstimulus timing in each sequence was the same between the two experiments. Subjects were again cued to attend a single sequence, either to the left or right, while ignoring irrelevant stimuli in the other two locations. Note that here, as shown in Figure 1, the cue onset came on 1 s before the first arrow onset, which was in the center distractor sequence. This is in contrast to Experiment 1, where the cue came on roughly the same amount of time before the first onset in the left arrow sequence. In Experiment 2, subjects completed 180 trials.

#### 2.2.3. Experiment 3

In Experiment 3, we again increased the number of stimuli. Eight arrows appeared on each trial, equally spaced around an invisible circle approximately 4.8° from central fixation (Figure 1A). The potential targets were again directly left and right of fixation, with the left sequence always leading the right sequence. The timing of these two sequences was identical to that of Experiment 2. The remaining distractor arrows, at the ±45° diagonal locations and above and below fixation, offset and onset in pairs once during the trial. That is, for example, at time 0.0 s, the upper left and lower right arrows onset, at time 1.0 s, the upper right and lower left arrows onset, and at time 2.0 s the upper and lower central arrows onset. Distractor arrows were always paired with their counterpart 180° across the display; the order in which these pairs onset was randomized on each trial. In Experiment 3, subjects completed 180 trials.

### 2.3. Data Collection

A 64-channel cap (BioSemi, Amsterdam, Netherlands), with electrode locations arranged according to the international 10–20 system, was used for EEG measurement. Two reference electrodes were placed on the mastoids in addition to three electrodes around the eyes for electrooculogram (EOG) measurement.

Stimuli were displayed on an LCD monitor in a sound-treated booth. During the course of the experiment, subjects were instructed to keep eyes open and fixate on a central fixation point, and to try to refrain from blinking during stimulus presentation. Behavioral data were collected in MATLAB while EEG was simultaneously recorded at 2,048 Hz using BioSemi ActiveTwo system hardware and its ActiveView data acquisition software. Tucker-Davis Technologies System 3 (TDT, Alachua, FL) hardware driven by MATLAB software generated triggers corresponding to stimulus and response events.

### 2.4. Data Analysis

#### 2.4.1. EEG Pre-processing

EEG data were processed using the EEGLAB toolbox for MATLAB (Delorme and Makeig, 2004). First, raw EEG data were re-referenced to the average between two mastoid electrodes and downsampled to 256 Hz. An FIR zero-phase filter was then applied with cutoffs at 0.5 and 50 Hz to remove slow drift and high-frequency noise from the signal. Eyeblinks were removed using independent component analysis (Hoffmann and Falkenstein, 2008), and trials with amplitudes exceeding ±100 μV were rejected. Trials in which subjects gave an incorrect response were also discarded before further analysis. CSD Toolbox (Kayser and Tenke, 2006) was used to transform the data from voltage to current source density, as this has been shown to reduce spatially correlated EEG noise (Kayser and Tenke, 2015; McFarland, 2015), which is desirable when localizing alpha power across the scalp.

#### 2.4.2. Event-Related Potential

To estimate the evoked response, or ERP, a bootstrap procedure was used as in Dai et al. (2018). First, the average response was calculated across 100 randomly chosen trials with replacement within a single subject and condition. This procedure was repeated 200 times. The estimated ERP for each subject and condition was taken as the average across these bootstrapped samples. The cue-evoked N1 was defined as the largest negative value of the ERP in a window between 75 and 240 ms following cue onset. We normalized each subject's ERP by computing the mean amplitude, across all channels, of the N1 response elicited by attend-left and attend-right cues. The entire ERP time course was then divided by this value. This normalization step compensated for individual differences in signal strength, ensuring that results were similar in magnitude across subjects. Grand averages were obtained for each experiment by averaging the normalized ERP amplitudes across subjects in each condition.

To quantify N1 amplitudes, normalized ERPs were first averaged across 2 clusters in 8 parietal-occipital (PO) channels in right (P2, P4, P6, P8, P10, PO4, PO8, O2) and left (P1, P3, P5, P7, P9, PO3, PO7, O1) hemispheres. Because visual stimuli are primarily represented in the contralateral hemisphere, neural responses to left arrow onsets were measured in right PO channels while neural responses to right arrow onsets were measured in left PO channels. In order to estimate N1 timings for each arrow onset, we generated grand average left PO and right PO normalized ERPs and selected the time with the largest negative value in a window between 75 and 240 ms following each stimulus onset. Each subject's ERP was visually inspected to confirm that N1s were correctly identified by this approach. Then, for each subject, we computed the average ERP amplitude across the relevant PO channels during a 50-ms window centered on each of these N1 time points.

To quantify attentional modulation of the N1 for each subject, an attentional modulation index, AMI_N1_, was calculated as:

(1) $$\begin{array}{l} \text{AM}{\text{I}}_{\text{N}1}=\text{N}1_{\text{attend}}-\text{N}1_{\text{ignore}} \end{array}$$

In this equation, N1_attend_ is the negative of the ERP amplitude elicited by a particular arrow onset in the attended location at the determined N1 time; N1_ignore_ is the negative of the ERP amplitude elicited at the same time by this arrow onset when it was ignored. Note that these estimated N1 amplitudes were multiplied by −1 first so that positive values of AMI_N1_ indicated N1s were overall larger in response to attended stimuli compared to ignored stimuli, as expected a priori. AMI_N1_ was calculated for each arrow onset in both left and right sequences and averaged for an overall AMI_N1_ measure. The N1 to the first left onset was excluded in these calculations since it elicited a strong automatic response regardless of cue condition, consistent with Choi et al. (2014).

#### 2.4.3. Induced Alpha Power

To obtain the induced alpha response, it was necessary to first remove phase-locked, or evoked activity. For each trial, each subject's average ERP for that condition was subtracted from the trial's time course to isolate the induced, or non-phase-locked, activity for each trial, as described in Kalcher and Pfurtscheller (1995). A short-time Fourier transform was then applied to each trial to estimate the power at each frequency in the alpha band (8–14 Hz). For each subject, their individual alpha frequency was determined by finding the frequency in the range of 8–14 Hz whose magnitude was largest in 20 PO channels. Once an individual's alpha frequency was selected, power was extracted at this frequency to produce a single time series for each trial in each EEG channel. The bootstrapping procedure described above was used to estimate each subject's average induced alpha power in each attention condition. These trial-averaged time series were then normalized for each subject by dividing each time point by the average alpha power across time, sensors, and experimental conditions. Grand averages were obtained from these normalized time series.

An attentional modulation index of alpha power, or Alpha Lateralization Index, ALI, was also quantified for each subject. Calculation of ALI is given by Equation (2). Here, α_ipsi_ is the average alpha power, collapsed across left and right PO sensors when subjects were attending to the ipsilateral sequence (i.e., *ignoring* the contralateral sequence). That is, we averaged together attend-left trials from left PO sensors and attend-right trials from right PO sensors. Similarly, α_contra_ is the average alpha power, collapsed across left and right PO sensors, when subjects were attending to the contralateral sequence (i.e., ignoring the ipsilateral sequence). That is, we averaged together attend-right trials from left PO sensors and attend-left trials from right PO sensors. For this measure, alpha was averaged over the stimulus period, which was defined as 0.6–3 s for Experiment 1 and 0.6–2.4 s for Experiments 2 and 3 (Figure 1A). Large positive values of ALI indicate that alpha power was overall larger in the ipsilateral attention condition (i.e., the alpha response was larger when subjects were ignoring the contralateral sequence relative to when that same sequence was being attended). Averages were calculated across the same left and right PO channels used for obtaining N1 amplitudes.

(2) $$\begin{array}{l} \text{ALI}=\frac{\alpha_{\text{ipsi}}-\alpha_{\text{contra}}}{\alpha_{\text{ipsi}}+\alpha_{\text{contra}}} \end{array}$$

#### 2.4.4. Significance Testing

To test for significant differences between N1s to attended and ignored stimuli, we used a permutation test as described in Maris and Oostenveld (2007). For each subject, the average N1 amplitude to attended stimuli was calculated across both left and right onsets as described above (N1_attend_, Equation 1). Similarly, the average N1 amplitude was calculated when subjects were told to ignore the same stimuli (N1_ignore_, Equation 1). A paired sample *t*-value was calculated from N1_attend_ and N1_ignore_. Values for N1_attend_ and N1_ignore_ were then swapped within-subject for all 2^*k*^ permutations, where *k* is the number of subjects. Since we hypothesized that the average N1 amplitude was greater in response to attended stimuli (N1_attend_) than when those same stimuli were being ignored (N1_ignore_), a one-sided test was used to determine if differences were significant.

We also tested whether this N1 modulation (N1_attend_ − N1_ignore_) was significant at each individual onset. For this purpose, we used a Wilcoxon signed rank test. Bonferroni-Holm correction was applied to correct for multiple comparisons.

Significance testing of alpha power differences was also conducted using a permutation test (Maris and Oostenveld, 2007). The mean alpha power in ipsilateral and contralateral attention conditions was calculated as in Equation (2) (α_contra_ and α_ipsi_). Paired *t*-values were then calculated as above for all 2^*k*^ permutations of α_contra_ and α_ipsi_ swapped within-subject. Since we hypothesized that the average alpha power was greater when stimuli were being ignored (α_ipsi_) than when those same stimuli were being attended (α_contra_), a one-sided test was used to determine if differences were significant.

The attentional modulation indices, AMI_N1_ and ALI, were compared across the three experiments. A Kruskal-Wallis one-way ANOVA was used to test for significant differences in these attentional modulation measures among the three tasks. This test was chosen instead of a one-way ANOVA since the ANOVA relies upon an assumption of normality, and we could not guarantee that this requirement was met by the small data sets compared here. *Post hoc* analyses were performed using the Mann-Whitney test for pairwise comparisons between paradigms. Multiple comparisons corrections was performed on these *post-hoc* tests using the Bonferroni-Holm method.

#### 2.4.5. EOG

Visual inspection of EOG data revealed that some subjects consistently saccaded in the direction of the cued location. Efforts were therefore made to quantify the degree of horizontal eye movement for each subject and to test whether this value was related to the degree of N1 or alpha modulation to ensure that any effects observed were not explained by eye movement. Horizontal EOG was obtained by taking the difference between electrodes placed on the left and right temples. EOG was then smoothed using a moving average filter and any linear trends were removed. The median EOG value of each trial was subtracted from all time points in the trial before quantifying saccades.

For each subject and attention condition, we then considered the distribution of horizontal EOG values across all trials. Leftward saccades resulted in more negative values within this distribution; rightward saccades resulted in more positive values. Thus, we took the mean absolute magnitude of the lowest 25% of EOG values in this distribution as the measure of leftwards saccades, S_L_, and the mean absolute magnitude of the highest 25% of EOG values in this distribution as the measure of rightwards saccades, S_R_. Then, we computed the difference in those magnitudes between attend-left (att_left) and attend-right (att_right) trials. The saccade index, SI, was calculated as shown in Equation (3), and gives the overall difference in saccades between attend-left and attend-right trials.

(3) $$\begin{array}{l} \text{SI}=\frac{{\text{S}}_{{\text{L}}_{\text{att\_left}}}-{\text{S}}_{{\text{L}}_{\text{att\_right}}}}{{\text{S}}_{{\text{L}}_{\text{att\_left}}}+{\text{S}}_{{\text{L}}_{\text{att\_right}}}}+\frac{{\text{S}}_{{\text{R}}_{\text{att\_right}}}-{\text{S}}_{{\text{R}}_{\text{att\_left}}}}{{\text{S}}_{{\text{R}}_{\text{att\_right}}}+{\text{S}}_{{\text{R}}_{\text{att\_left}}}} \end{array}$$

For each subject, AMI_N1_ and ALI were calculated as in Equations (1) and (2) and compared to SI. Spearman's rank-order correlation coefficients were calculated to measure the strength of the relationship between SI and both AMI_N1_ and ALI. A strong positive correlation would suggest that modulation of the N1 and alpha power could be explained by eye movements.

#### 2.4.6. Passive Trials

Data from passive trials are not reported here due to differences in EOG data between these trials and attend-left or attend-right trials. Most notably, we observed more blinks during passive trials, which means that subjects may have spent more time during these trials with eyes closed. Alpha oscillations are strongly elicited when eyes are closed, so we could not rule out that differences in alpha power between attend and passive conditions were simply due to differences in eye activity. No differences in EOG data contributed to differences in alpha for leftward vs. rightward attention in any of the three paradigms.

## 3. Results

### 3.1. Behavior

#### 3.1.1. Performance on All Three Tasks Was at Ceiling

Performance was measured as percent correct response for attend-left and attend-right trials. The mean percent correct for attend-left trials was 93.3 (*SD* = 5.3), 96.4 (*SD* = 5.5), and 93.8 (*SD* = 6.8) for Experiments 1, 2, and 3, respectively. The mean percent correct for attend-right trials was 93.5 (*SD* = 4.4), 95.5 (*SD* = 4.9), and 94.0 (*SD* = 5.2) for Experiments 1, 2, and 3, respectively. These near-ceiling performance measures indicate that subjects could successfully perform the task in each experiment and condition. No significant differences in percent correct response were found between different experiments or between attend-left and attend-right trials (*p* > 0.2 for all comparisons).

### 3.2. EEG

#### 3.2.1. In Experiment 1, Spatial Attention Amplified the N1 Response Contralateral to Attended Stimuli and Increased Alpha Power Contralateral to Ignored Stimuli

**Figure 2:**
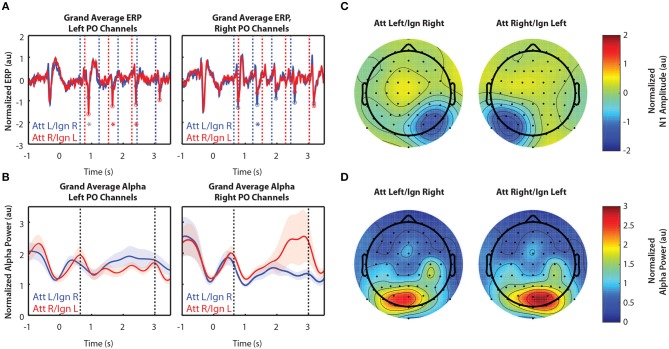
Grand average normalized ERP and alpha power for Experiment 1. All error bars represent the standard error of the mean. **(A)** Time course of the grand-averaged ERP, averaged across left parietal-occipital channels (left) and right parietal-occipital channels (right). Red and blue vertical lines indicate right and left arrow onset times, respectively. Red and blue circles indicate points at which N1 amplitudes were measured for attend-right and attend-left trials, respectively. Asterisks indicate significant differences in N1 amplitude between “attend” and “ignore” conditions (signed rank test; *p* < 0.05). Gray asterisks indicate comparisons that did not remain significant after Bonferroni-Holm correction. **(B)** Time course of the grand-averaged alpha power, averaged across left parietal-occipital channels (left) and right parietal-occipital channels (right). Vertical black lines denote the beginning and end of stimulus presentation. Note that normalized alpha power values of 1 correspond to baseline alpha levels, as determined by the average across trials, sensors, and time. **(C)** Spatial distribution of the N1 for Experiment 1. Attend-left and attend-right N1s were averaged across left and right stimulus onsets, respectively. **(D)** Spatial distribution of alpha power, averaged across the entire stimulus period for both attend-left and attend-right conditions.

Figure 2 shows grand-averaged (*n* = 10) EEG data for Experiment 1. Recall that Experiment 1 contained only two arrows, one to the left and one to the right of a central fixation point. Figure 2A shows the time course of ERPs in left and right PO channels. Note that strong responses to right stimuli occur in left PO channels, while strong responses to left stimuli occur in right PO channels. Consistent with our expectations, in left PO channels (Figure 2A, left), N1 responses were larger to right stimuli in attend-right trials (red circles) than in attend-left trials (blue circles). Conversely, in right PO channels (Figure 2A, right), N1 responses were larger to left stimuli in attend-left trials than in attend-right trials.

Alpha power was greater over PO channels ipsilateral to the attended location, which primarily represent ignored stimuli. This is shown in Figure 2B, where alpha power remains higher throughout the stimulus period when a given stimulus was ignored (blue trace in left PO channels and red trace in right PO channels) compared to when it was attended. These results show that modulation of alpha power persists during sustained attention to ongoing stimuli. Note that no differences in alpha between cue-left and cue-right trials were observed in the period of time following the cue but preceding stimulus presentation (−0.5–0.6 s). Thus, there appears to be no anticipatory biasing of attention preceding the stimulus period in Experiment 1, which used relatively simple stimuli.

Figure 2C shows the spatial distribution of N1 amplitudes averaged across the time points indicated by the circles in Figure 2A. For both cue-left and cue-right trials, we saw a strong N1 response contralateral to the attended stimuli, demonstrating the contralateral specificity of the N1 response. We found that alpha power was also strongly lateralized, as displayed in Figure 2D. Here, alpha power was averaged across the entire stimulus period (0.6–3 s). We see that power was greater in channels contralateral to ignored stimuli. This is consistent with the hypothesis that alpha power reflects the suppression of *ignored* stimuli, suggesting that both selection of targets *and* suppression of distractors play a role during sustained top-down control of spatial attention.

#### 3.2.2. In Experiment 2, Attentional Modulation of the N1 and Alpha Power Was Similar to That of Experiment 1

**Figure 3:**
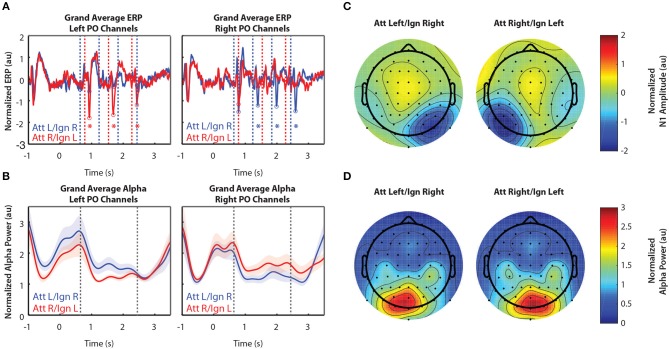
Grand average normalized ERP and alpha power for Experiment 2. All error bars represent the standard error of the mean. **(A)** Time course of the grand-averaged ERP, averaged across left parietal-occipital channels (left) and right parietal-occipital channels (right). Red and blue vertical lines indicate right and left arrow onset times, respectively. Red and blue circles indicate points at which N1 amplitudes were measured for attend-right and attend-left trials, respectively. Asterisks indicate significant differences in N1 amplitude between “attend” and “ignore” conditions (signed rank test; *p* < 0.05). Gray asterisks indicate comparisons that did not remain significant after Bonferroni-Holm correction. **(B)** Time course of the grand-averaged alpha power, averaged across left parietal-occipital channels (left) and right parietal-occipital channels (right). Vertical black lines denote the beginning and end of stimulus presentation. Note that normalized alpha power values of 1 correspond to baseline alpha levels, as determined by the average across trials, sensors, and time. **(C)** Spatial distribution of the N1 for Experiment 2. Attend-left and attend-right N1s were averaged across left and right stimulus onsets, respectively. **(D)** Spatial distribution of alpha power, averaged across the entire stimulus period for both attend-left and attend-right conditions.

Figure 3 shows grand-averaged EEG data (*n* = 11) for Experiment 2. Recall that Experiment 2 was the same as Experiment 1, except that in addition to the left and right arrows, another distractor was presented above fixation. Arrows appearing in that location were always ignored. Figures 3A,B show the time course of the grand-averaged ERP and alpha power, respectively. These results are similar to those observed for Experiment 1. N1s were consistently larger when the eliciting stimuli were attended compared to when they were ignored, though this modulation appears larger than that of Experiment 1. Like Experiment 1, alpha power was also larger in response to ignored stimuli. The spatial distribution of the N1 and alpha power were also similar to that observed in Experiment 1. As in Experiment 1, while the N1 enhancement occurred in sensors representing attended stimuli (Figure 3C), alpha power increased over sensors representing ignored stimuli (Figure 3D). One difference from Experiment 1, though, is that lateralization of alpha power seems to appear after the cue for where to attend and before the arrows are presented, in anticipation of the upcoming stimuli (−0.5–0.6 s and −1–0 s for Experiments 1 and 2, respectively). A two-sided Mann-Whitney U-test confirmed that this lateralization, measured by ALI, was significantly different between Experiments 1and 2 (*U* = 79, *p* = 0.031). Note that alpha power also appears overall higher in both conditions after the stimulus period for Experiment 2 than Experiment 1, but this is likely due to the fact that more of the response period is displayed in Figure 3B than in Figure 2B. Pre-stimulus alpha power also appears higher overall in Experiment 2 than in Experiment 1, though further experiments with a larger number of subjects would be needed to determine whether or not these differences could be explained by variation in subjects across experiments, since estimates of alpha power tend to be noisy.

#### 3.2.3. In Experiment 3, Spatial Attention Amplified the N1 Response Contralateral to Attended Stimuli, but Did Not Appear to Modulate Alpha Power

**Figure 4:**
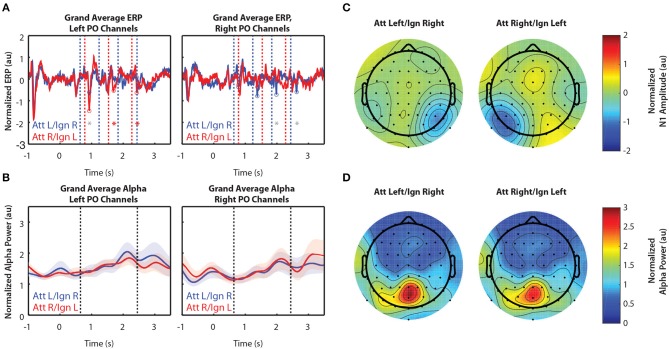
Grand average normalized ERP and alpha power for Experiment 3. All error bars represent the standard error of the mean. **(A)** Time course of the grand-averaged ERP, averaged across left parietal-occipital channels (left) and right parietal-occipital channels (right). Red and blue vertical lines indicate right and left arrow onset times, respectively. Red and blue circles indicate points at which N1 amplitudes were measured for attend-right and attend-left trials, respectively. Asterisks indicate significant differences in N1 amplitude between “attend” and “ignore” conditions (signed rank test; *p* < 0.05). Gray asterisks indicate comparisons that did not remain significant after Bonferroni-Holm correction. **(B)** Time course of the grand-averaged alpha power, averaged across left parietal-occipital channels (left) and right parietal-occipital channels (right). Vertical black lines denote the beginning and end of stimulus presentation. Note that normalized alpha power values of 1 correspond to baseline alpha levels, as determined by the average across trials, sensors, and time. **(C)** Spatial distribution of the N1 for Experiment 3. Attend-left and attend-right N1s were averaged across left and right stimulus onsets, respectively. **(D)** Spatial distribution of alpha power, averaged across the entire stimulus period for both attend-left and attend-right conditions.

Figure 4 shows grand-averaged (*n* = 10) EEG data for Experiment 3. In contrast to Experiments 1 and 2, Experiment 3 had more salient distractor stimuli, as arrows flashed in a total of 8 locations during the stimulus period while attention was to be directed only to locations left or right of a central fixation point. Figure 4A still shows clear N1 amplification in response to attended stimuli, but N1 amplitudes were overall weaker than those observed in Experiments 1 and 2. In addition, we observed no difference in alpha power between attend-left and attend-right trials, either within a single group of sensors (Figure 4B) or across all 64 sensors (Figure 4D).

#### 3.2.4. Modulation of EEG Reflected Enhancement of Attended Stimuli and Suppression of Ignored Stimuli in Experiments 1 and 2, but Only Enhancement of Attended Stimuli Was Observed in Experiment 3

**Figure 5:**
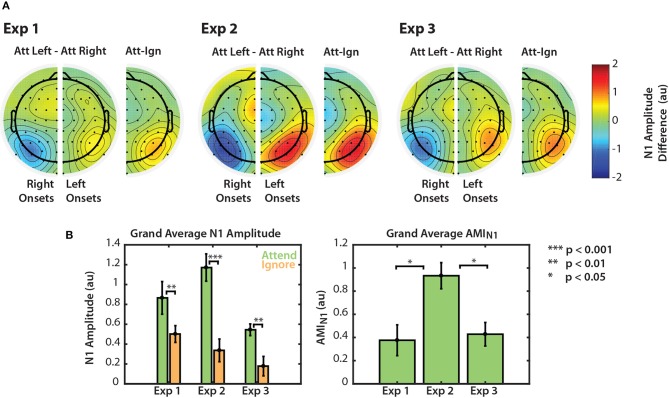
Normalized N1 amplitude modulation summarized for all three experiments. **(A)** Differences in N1 amplitude between attend-left and attend-right trials. N1 differences for left and right onsets are represented separately in right and left PO channels, respectively. As noted in section 2.4.2, the first left onset was not included in this calculation. Differences are also represented on one half of the scalp as the difference between ipsilateral and contralateral attention conditions. **(B)** Grand average N1 amplitudes to attended and ignored stimuli (left) and AMI_N1_ (right). Error bars represent the standard error of the mean.

Modulation of the N1 is summarized for the three visual tasks in Figure 5. Figure 5A shows grand average differences in normalized N1 amplitude between attend and ignore conditions. For each of Experiments 1–3, we computed the difference in N1 amplitude between attend-left and attend-right trials, for both right arrow onsets (left channels) and left arrow onsets (right channels). Then, we collapsed across the midline to show an overall difference between “attend” and “ignore” conditions, projected onto right channels. Across all three experiments, N1 amplitudes in PO channels were consistently larger to particular stimuli when those stimuli were attended compared to when they were ignored.

Normalized N1 amplitudes to attended and ignored stimuli are shown in the left panel of Figure 5B. For all three experiments, the average N1 response to attended stimuli was larger than the response to ignored stimuli. A permutation test confirmed that these amplitude differences were significant (permutation test; *p* = 0.0039, *p* = 0.00048, *p* = 0.0020 for Experiments 1, 2, and 3, respectively. See Figure 5B, left panel). This result provides evidence that top-down spatial attention is sustained throughout stimulus presentation regardless of scene complexity. Note, however, that this statistical significance did not hold for all individual N1s across the three experiments (see Figures 2A, 3A, 4A). N1 amplitude differences between “attend” and “ignore” conditions were significant across all onsets in Experiment 2, but not in Experiments 1 and 3. This is likely due to the fact that we do not have the statistical power to observe this modulation at the single onset level in these experiments. Nonetheless, when we averaged the differences between “attend” and “ignore” conditions across onsets (AMI_N1_), we found that the overall modulation during stimulus presentation was significantly greater than zero in all three experiments (Mann-Whitney, *p* < 0.01 for all comparisons). These N1 attention modulation indices are shown in the right panel of Figure 5B. Note that although absolute N1 amplitudes appeared to be smaller for Experiment 3 (left panel), the mean modulation index was similar to that of N1s in Experiment 1 (right panel). The average AMI_N1_ in Experiment 2 appeared to be much larger than those in Experiments 1 and 3, and a Kruskall-Wallis one-way ANOVA found a significant difference in AMI_N1_ among the three paradigms [$\chi_{\left(2\right)}^{2}=10,p=0.0067$]. A Mann-Whitney test indicated that AMI_N1_ was significantly larger for Experiment 2 than for Experiments 1 (*U* = 73, *p* = 0.0203, after corrections for 3 comparisons) and 3 (*U* = 160, *p* = 0.0201, after correction for 3 comparisons). No significant difference in AMI_N1_ was found between Experiments 1 and 3.

**Figure 6:**
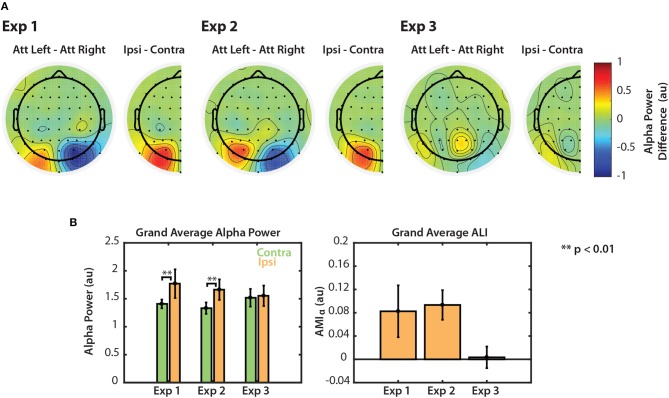
Normalized alpha power modulation summarized for all three experiments. **(A)** Differences in alpha power between attend-left and attend-right trials. Differences are also shown collapsed across hemispheres as the general difference between ipsilateral and contralateral attention conditions. **(B)** Grand average alpha ipsilateral and contralateral to the attended sequence (left) and ALI. Error bars represent the standard error of the mean.

Modulation of alpha power is summarized for the three visual tasks in Figure 6. Figure 6A shows grand average differences in normalized alpha power between attend-left and attend-right trials, averaged across the stimulus period (0.6–3 s for Experiment 1 and 0.6–2.4 s for Experiments 2 and 3). We chose to collapse across the entire stimulus period since we observed that these alpha differences were sustained during this period of time (see Figures 2B, 3B, 4B). For each experiment, the difference in alpha power between attend-left and attend-right trials is shown at each channel. We again collapsed across the midline to show the difference in alpha power between attend-ipsilateral trials and attend-contralateral trials, and projected these values onto right channels for visualization. For Experiments 1 and 2, we saw that alpha power was greater in PO channels when attending to a target in the ipsilateral hemifield than when attending to a target in the contralteral hemifield. In Experiment 3 we saw no such effects.

The left panel of Figure 6B shows grand average alpha power for each experiment, collapsed across attend-left and attend-right conditions. In Experiments 1 and 2, average alpha power was significantly greater when subjects attended the ipsilateral (ignored contralateral) sequence than when they attended the contralateral sequence (permutation test; *p* < 0.01). In Experiment 3, however, no significant difference was observed (permutation test; *p* = 0.287). Alpha lateralization index, ALI is shown in the right panel of Figure 6B. ALI for Experiments 1 and 2 was larger than for Experiment 3. A Kruskal-Wallis one-way ANOVA indicated that there were small differences in ALI between the three tasks [χ^2^(2) = 6.296, *p* = 0.0429]. No significant difference was found in ALI between Experiments 1 and 2 (*U* = 93, *p* = 0.2453, after correction for 3 comparisons) or between Experiments 1 and 3 (*U* = 126, *p* = 0.2422, after correction for 3 comparisons). While ALI was larger in Experiment 2 than in Experiment 3, this difference was not significantly different after correction for multiple comparisons (*U* = 154, *p* = 0.0663, corrected for 3 comparisons). This result is likely due to the overall low statistical power in this study. Nonetheless, these results suggest that top-down control of visual selective attention results in modulation of both the N1 and alpha power. However, if the scene is more complex, as in Experiment 3, top-down control is no longer represented by clear modulation of alpha power across left and right PO channels.

#### 3.2.5. Neither N1 nor Alpha Modulation Correlated With Behavioral Measures

We also examined individual subject data to test whether the degree of N1 amplitude modulation or of alpha power modulation was correlated with performance on the task. Neither the average difference in N1 amplitude nor the average difference in alpha power between attend-left and attend-right conditions in a single group of sensors (left or right PO), or collapsed across sensors (ALI) was correlated with percent correct response for any of the three experiments. Of course, given that performance was near ceiling in all cases, this null result may simply reflect the lack of variability in our behavioral measures.

### 3.3. EOG

#### 3.3.1. Differences in N1 and Alpha Power Modulation Cannot Be Explained by Eye Movement

Unfortunately, most of the EOG data for Experiment 1 could not be analyzed due to a recording error. However, EOG data from Experiments 2 and 3 were robust, which allowed us to examine potential relationships between EOG and EEG measures. Upon visual inspection, in both experiments, subjects that had a large saccade index (SI) consistently saccaded in the direction of the target during the stimulus period, where those with SI close to zero showed no discernable difference in EOG between attend-left and attend-right trials. We found no significant difference in SI (*U* = 118, *p* = 0.8603) between Experiment 2 (mean = 0.5785, *SD* = 0.6789, min = −0.0227, max = 2.1790) and Experiment 3 (mean = 0.5665, *SD* = 0.6148, min = −0.2137, max = 1.4114).

For Experiment 2, there was no significant correlation found between saccade index (SI) and AMI_N1_ [ρ(9) = 0.1, *p* = 0.778]. However, when comparing EOG measures to alpha modulation, we found that SI had a strong negative correlation with ALI [ρ(9) = −0.85, *p* = 0.0016]. In other words, subjects who saccaded in the direction of the target—producing larger values of SI—displayed little, if any, alpha modulation in EEG throughout the trial. These results are intuitive since subjects who fixated on the target most likely could rely on the enhanced representation of foveated (vs. peripheral) stimuli. Therefore, if eye movements did occur during the task, they would only reduce any alpha modulation measured in the EEG signal; the eye movements thus do not explain the alpha results. In Experiment 3, no strong correlations were found between the SI and ALI [ρ(8) = −0.14, *p* = 0.707]. This is most likely due to the fact that values of ALI were all close to zero in Experiment 3. Since SI was not significantly different between Experiments 2 and 3, the lack of alpha modulation observed in Experiment 3 is not likely due to excessive eye movement. We did find a positive correlation between SI and AMI_N1_ in Experiment 3, however [ρ(8) = 0.697, *p* = 0.031], suggesting that the subjects who made large saccades during stimulus presentation had greater N1 modulation. This makes sense given the amount of stimulus clutter in Experiment 3; relative to those who covertly attended, subjects who foveated on the target likely had greater N1 modulation due to the competing stimuli being outside the field of view. The fact that Experiment 2 had a low amount of stimulus clutter may explain why no correlation between AMI_N1_ and SI was observed for this task, as the relative difference in N1 modulation between those who overtly and covertly attended was likely smaller. Furthermore, that AMI_N1_ was actually greater in Experiment 2 than in Experiment 3 suggests that this correlation does not explain the N1 modulation differences between experiments observed here.

## 4. Discussion

### 4.1. In Simple Visual Scenes, Attention Modulates Both the N1 and Alpha Power During Presentation of Dynamic Stimuli

The visual task required subjects to not only shift attention to a single location, but also track how the object in that location changed over time. This allowed us to explore how the N1 and alpha power reflect attention during presentation of dynamic stimuli (as opposed to just before or just after a single static object is presented).

As expected, attention modulated the N1 response to each arrow onset, amplifying the response in EEG sensors contralateral to the location of the target stimuli. Previous studies using an analogous auditory task (Choi et al., 2013, 2014; Dai et al., 2018) found that, compared to passive listening, the amplitude of the N1 to tone onsets in a melody was enhanced when the tones were attended and inhibited when they were ignored. Thus, our N1 results are consistent, not only with previous visual studies (Mangun and Hillyard, 1991; Hillyard and Anllo-Vento, 1998), but also with those obtained during a similar auditory task.

Previous studies have shown that attention modulates alpha: alpha power is generally greater over cortical regions processing stimuli in an ignored location (that is, regions that are ipsilateral to the attended location) (Kelly et al., 2006; Foxe and Snyder, 2011). This has frequently been shown to occur after a cue indicating what to attend (and before the stimulus appears) (Worden et al., 2000; Thut et al., 2006), and just after a brief presentation of a single stimulus (Sauseng et al., 2005; van Diepen et al., 2016). It should follow that if subjects are required to continuously suppress a sequence of distractor stimuli that alpha modulation should be continuously engaged during presentation of those stimuli. Indeed, this has been shown when subjects are required to count target letters in one of two bilaterally presented sequences (Kelly et al., 2006) and when subjects track continuous movement of dots in one of two bilateral arrays (Händel et al., 2011). Our results expand on these finding by providing evidence that alpha modulation reflects a suppression mechanism during sustained spatial attention to strong, dynamic visual input. By calculating the induced alpha response, we were able to isolate neural activity that was not a result of the evoked response to flashing stimuli. The fact that the N1 was modulated across the sequence of arrows indicates that top-down control was directed toward cued locations throughout the stimulus period. Alpha modulation occurred simultaneously with N1 modulation, with power was greater over cortical regions corresponding to the ignored locations. These results support the idea that alpha reflects a suppression of irrelevant information during selection of relevant information.

### 4.2. The Complexity of the Scene Affects the Strength of the N1 and Alpha Power Modulation Measured Using EEG

All three experiments had the same basic task: attend arrows making up either the left or right sequence and report their orientation trajectory. Behavioral data suggest that this task, regardless of scene complexity, was not particularly difficult—target trajectories were correctly identified on 94% of all trials across experiments. However, EEG markers of top-down spatial attention differed with the degree of irrelevant stimulus clutter that participants needed to ignore. This suggests that although performance did not vary meaningfully, top-down attention mechanisms were nonetheless modulated by the complexity of the scene.

The results show that in simple scenes (e.g., Experiments 1 and 2), top-down control of attention is reflected in EEG by both amplification of the N1 response to targets and greater alpha power over cortices processing to-be-ignored locations. However, in a more complex scene, such as that of Experiment 3, the neural representation of spatial attention is more difficult to interpret. The N1 response was still modulated in response to attended stimuli, but the amplitudes were overall smaller than those observed in the other two paradigms. In addition, the spatial distribution of alpha power was not lateralized across PO channels during stimulus presentation as it was in Experiments 1 and 2. Even within a single group of sensors on the left or right, spatial attention did not appear to modulate alpha power over time.

One way to interpret the lack of alpha modulation observed in Experiment 3 data is that due to the complexity of the scene, more suppression of irrelevant stimuli must be performed by the brain. Unlike Experiment 2, Experiment 3 presented distracting stimuli in both the left and right visual fields. Therefore, while attending the target sequence on the left, for example, the subject had to suppress the competing sequence to the right, the distractors to the right, and the distractors in the left portion of the display. If we assume that suppression of spatial attention operates in a very precise, retinotopically specific manner (Worden et al., 2000; Kelly et al., 2006; Payne and Sekuler, 2014), then alpha power would increase in left parietal-occipital cortex to suppress all distractors on the right. However, alpha power would *also* have to increase in right PO cortex to suppress distracting stimuli on the left. Due to the limited spatial resolution of EEG, this may mask any spatial effects on alpha power in these brain regions when the scene is complex, with distactors in both hemifields.

The overall smaller N1s in Experiment 3 are consistent with this account. It may be that alpha suppression of objects in the left portion of the scene spills over, partially attenuating the neural response to nearby targets. Since they are still targets, however, the N1 is still modulated, but with overall smaller amplitude. This is consistent with the theory that alpha-band oscillations gate information flow to object-selective cortex (Hanslmayr et al., 2013; Zumer et al., 2014). Alternatively, it may be that the overall smaller N1s in Experiment 3 were simply due to a more crowded visual scene (Chicherov et al., 2014). ERPs elicited by these distractors could have interfered destructively with ERPs elicited by targets, reducing average N1 amplitude on the scalp.

The fact that the EEG data for Experiments 1 and 2 were similar suggests that the scene complexity presented by these two paradigms was also similar. We did, however, observe alpha modulation that preceded stimulus presentation in Experiment 2, but not in Experiment 1. This indicates that anticipatory biasing of attention occurred during Experiment 2, perhaps as a result of adding a single distractor that came on before both the left and right sequences. By adding this distractor, stimulus presentation, though irrelevant, started earlier in Experiment 2 than in Experiment 1. This may have caused subjects to focus spatial attention earlier in the trial. Additionally, while the absolute N1 amplitudes were similar between Experiments 1 and 2, modulation of the N1 was significantly larger in Experiment 2 than in Experiment 1. One possibility for why this is the case may be due to the addition of the single center distractor in Experiment 2. While this distractor may not have added substantial spatial complexity to the scene, since it was always in a single location, it was an additional stimulus to suppress, which may have contributed to greater N1 modulation. The fact that alpha modulation during stimulus presentation was similar between the two paradigms—but absent in Experiment 3—supports this explanation, since the degree of alpha lateralization seems to most closely index the degree of spatial complexity in the scene.

Though Experiments 2 and 3 both had always-irrelevant stimuli, alpha modulation was not degraded in Experiment 2. This is probably due to the simplicity of distractors in Experiment 2. Unlike Experiment 3, Experiment 2 had a single distractor placed in the center of the display, just above the central fixation point. This distractor may have been far enough away from either target sequence that when focused attention was directed at the target, this distractor could be grouped with the competing sequence. In other words, an increase in alpha power over just one hemisphere may have been enough to suppress the center distractor. It is also possible that because the irrelevant center sequence was predictable and always ignored, that the brain did not employ alpha oscillations to suppress it at all. Such an effect would result in only having to suppress the competing arrow sequence. This would most likely not be the case in Experiment 3 since the order in which always-ignored arrows flashed was randomized and therefore unpredictable. Subjects could therefore not learn to ignore them as easily. An alternative to these explanations, however, is that center stimuli did not contribute at all to lateralized alpha power measures in either Experiment 2 or 3, as previous studies have demonstrated that stimuli at midline do not elicit lateralized evoked EEG responses (Hickey et al., 2009). Therefore, alpha lateralization differences between Experiments 2 and 3 could be based solely on the left and right distractors added in Experiment 3. This would also explain the similar alpha lateralization observed in Experiments 1 and 2.

### 4.3. Limitations

We should note that in designing Experiments 1, 2, and 3, the complexity of the scene was only defined by the number of locations in which always-ignored stimuli were presented. Even working within this definition, we did not explore a large range of scene complexities in this study. Experiments 1 and 2 were similar, presenting relatively simple scenes that contained zero and one always-ignored stimulus locations, respectively. Experiment 3, however, presented a scene of much higher complexity with six always-ignored locations. Thus, scene complexity, as defined here, was similar in Experiments 1 and 2, but increased greatly from Experiment 2 to Experiment 3. In addition, stimuli at always-ignored locations in Experiment 3 were always on screen—each flashing off and on once each per trial—while the distractor stimuli in Experiment 2 flashed off and on three times at the same location. This could have contributed to differences in EEG observed over parietal-occiptial channels. Future work should be more rigorous in defining and testing the parameters that contribute to scene complexity.

It is also important to point out that the number of subjects analyzed for this study was low compared to most EEG studies. Individual subject measures of alpha power are noisy, and this fact, combined with the low sample sizes studied here, likely contributed to overall low statistical power. Additionally, we found that, in at least one experiment, saccades made by some subjects influenced the degree of N1 modulation observed, adding yet another confounding factor to our interpretation. If more subject data were collected, then subjects who made these large saccades could simply be excluded from analysis. Therefore, future work that aims to identify differences in N1 and parietal alpha power modulation across conditions should collect data from a larger number of subjects.

## 5. Conclusions

Obtaining non-invasive measures of spatial attention not only advances our understanding of how the brain parses a complex scene, but also provides a potential tool for monitoring attention in real time. If the scene is simple enough, our results suggest that both the N1 and alpha power could be used to decode the direction of spatial attention, even when stimuli in a particular location are not static. However, if there are many dynamic stimuli within a scene, then the neural representation of top-down control may be too complex to be discerned using EEG measures.

Even in the context of simple scenes, future work should be performed to examine the strength of N1 and alpha modulation within individual subjects to assess these measures as a viable tool for monitoring spatial attention. Previous findings have shown that single-trial auditory ERPs can be used to determine which objects in a mixture are selected (Choi et al., 2013), and visual ERPs are often used for the same purpose, for example in brain-computer interfaces (Thulasidas et al., 2006). Classifying direction of attention based on alpha modulation would prove even more powerful, however, since one would not need to know the exact timing of stimuli in the attended location.

We conclude that, in simple visual scenes, defined by few irrelevant stimuli, top-down control of visuospatial attention is represented in EEG by strong modulation of both the N1 and alpha power with the direction of attentional focus. This strong modulation occurs not only before stimuli are presented, but also during attention to strong dynamic visual input. However, as the scene becomes more complex, the neural representation of top-down control becomes more complex, reducing the strength of the observed attentional modulation of evoked and induced EEG signals.

## Data Availability Statement

The datasets generated for this study are available on request to the corresponding author.

## Ethics Statement

The studies involving human participants were reviewed and approved by Boston University Institutional Review Board. The participants provided written informed consent to participate in this study.

## Author Contributions

LB, SB, JK, and BS-C designed the experiments. LB, SB, and JK performed the experiments. LB, SB, JK, AN, and BS-C analyzed the data. LB, AN, and BS-C wrote the paper. BS-C was the principal investigator.

### Conflict of Interest

The authors declare that the research was conducted in the absence of any commercial or financial relationships that could be construed as a potential conflict of interest.
